# Genome-Wide Identification of *YABBY* Gene Family in Cucurbitaceae and Expression Analysis in Cucumber (*Cucumis sativus* L.)

**DOI:** 10.3390/genes13030467

**Published:** 2022-03-07

**Authors:** Shuai Yin, Sen Li, Yiming Gao, Ezra S. Bartholomew, Ruijia Wang, Hua Yang, Chang Liu, Xiaofeng Chen, Ying Wang, Xingwang Liu, Huazhong Ren

**Affiliations:** 1 Engineering Research Center of Breeding and Propagation of Horticultural Crops, Ministry on Education, College of Horticulture, China Agricultural University, Beijing 100193, China; yinshuaicau@163.com (S.Y.); s20203172667@cau.edu.cn (S.L.); gaoyiming@cau.edu.cn (Y.G.); ezrabartholomew@hotmail.com (E.S.B.); wangruijia@cau.edu.cn (R.W.); sy20213172882yang@cau.edu.cn (H.Y.); sy20213172844@cau.edu.cn (C.L.); liuxw01@cau.edu.cn (X.L.); 2 College of Ocean and Agricultural Engineering, Yantai Institute of China Agricultural University, Yantai 264670, China; cauyt2021@126.com; 3 Heze Agricultural and Rural Bureau, 1021 Shuanghe Road, Mudan District, Heze 274000, China; sannong2006@163.com; 4 Sanya Institute of China Agricultural University, Sanya 572000, China

**Keywords:** Cucurbitaceae, *YABBY* genes, evolution, expression pattern, cucumber

## Abstract

YABBY transcription factors play important roles in plant growth and development. However, little is known about *YABBY* genes in Cucurbitaceae. Here, we identified 59 *YABBY* genes from eight cucurbit species, including cucumber (*C*. *sativus* L.), melon (*C. melon* L.), watermelon (*C. lanatus*), wax gourd (*B. hispida*), pumpkin (*C. maxima*), zucchini (*C. pepo* L.), silver-seed gourd (*C. argyrosperma*), and bottle gourd (*L. siceraria*). The 59 *YABBY* genes were clustered into five subfamilies wherein the gene structures and motifs are conserved, suggesting similar functions within each subfamily. Different *YABBY* gene numbers in eight cucurbit species indicated that gene loss or duplication events exist in an evolutionary process across Cucurbitaceae. The *cis*-acting elements analysis implied that the *YABBYs* may be involved in plant development, and phytohormone, stress, and light responses. Importantly, *YABBY* genes exhibited organ-specific patterns in expression in cucumber. Furthermore, a gene *CsaV3_6G038650* was constitutively expressed at higher levels at different fruit development stages and might play a crucial role in cucumber fruit development. Collectively, our work will provide a better understanding for further function identifications of *YABBY* genes in Cucurbitaceae.

## 1. Introduction

The *YABBY* gene family, which belongs to the zinc finger protein superfamily, is plant-specific transcription factors (TFs). All YABBY members share two highly conserved domains that are characterized by a N-terminal C2C2 zinc finger domain and a helix–loop–helix motif (called the YABBY domain) at their C-terminus [1,2]. The first *YABBY* gene family was described and six members were identified in Arabidopsis, including *YABBY1* (*YAB1*)/*FILAMENTOUS FLOWER* (*FIL*), *CRABS CLAW* (*CRC*), *INNER NO OUTER* (*INO*), *YABBY2* (*YAB2*), *YABBY3* (*YAB3*), and *YABBY5* (*YAB5*) [1,2,3]. It has been reported that the *YABBY* genes are unique to seed plants [4,5] and genome-wide identification studies of the *YABBY* gene family have been performed in various plant species, such as tomato (9) [6], Chinese cabbage (12) [7], rice (8) [8], and maize (13) [9].

Based on evolutionary relationships, the angiosperm *YABBY* genes can be classified into five subfamilies—YAB1, CRC, INO, YAB2, and YAB5 [10,11]. Substantial evidence has demonstrated that the *YABBY* genes play important roles in many aspects of plant growth and development, such as lateral organ development, establishment of polarity, and reproductive organ development in angiosperms [12,13]. In Arabidopsis, four genes—*FIL*, *YAB2*, *YAB3*, and *YAB5*—are involved in vegetative tissues development [13,14,15]. *FIL*, *YAB2*, and *YAB3* are specifically expressed in all lateral organ primordia derived from the apical and flower meristems [2,14,16]. *FIL* and *YAB3* are responsible for leaf development in a redundant manner with *YAB2* and *YAB5* [2,14,17], and *FIL* also contributes to the establishment of floral meristem identity and flower development [2,16,18,19]. While *CRC* and *INO* regulate reproductive organ development [1,3,13]. *CRC* is required for carpel and nectary development [1,20,21], *INO* promotes the formation and asymmetric growth of ovule outer integument [3,22]. Furthermore, in monocots, the biological functions of *YABBY* genes have been well studied in rice. For example, *OsDL*, the homolog of *CRC*, affects the development of both flowers and leaves [23,24,25]. During rice domestication, the *YABBY* genes *OsSh1* and *ObSh3* are required for seed shattering [26,27]. *OsYAB1* controls meristem development and the maintenance of stamens and carpels [28]. *OsYAB3* (*TOB3*), *OsYAB4* (*TOB2*), and *OsYAB5* (*TOB1*) are enriched in lateral organ primordia and play a crucial role in rice spikelet development [29,30]. These results suggest that *YABBY* genes have diverse roles in plant growth and development; meanwhile, the functions of each subgroup are both differentiated and conserved.

The Cucurbitaceae are widely distributed in the tropics and subtropics [31]. The major cucurbit crops have global economic importance, such as cucumber (*C. sativus* L.), melon (*C. melo* L.), watermelon (*C. lanatus*), and squash/pumpkin (*C. maxima*) [31]. These cucurbit crops mainly consume their fruits. For example, cucumber and watermelon are consumed fresh fruits [32,33,34] and melon mainly contributes to diets as a sweet and fleshy dessert [35]. Many cucurbit crops contain important nutrients, such as sugars and lycopene in watermelon [36], and vitamins and flavonoids in wax gourd [37]. Importantly, the metabolites derived from cucurbit crops can function as medicines, for instance, the amino acids citrulline, arginine, and glutathione from watermelon promote cardiovascular health [38,39], the wax gourd metabolites can be used in treating various disorders [40,41], the cucurbitacins produced by cucurbit plants play a vital role in cancer therapy [42,43]. Although the *YABBY* gene family has been characterized in many plant species, little is known of *YABBY* gene characteristics in Cucurbitaceae. There are only two reports on the *YABBY* gene functions in cucumber. Liu et al. (2018) found that *CsYAB1*, *CsYAB3*, and *CsINO* are involved in the integument of ovules by interacting with *CsSPL* [44]. The other report suggested that *CsYAB5* regulates leaf morphology, and vascular and fruit development [45].

Here, we identified and characterized the *YABBY* genes in Cucurbitaceae, including cucumber (*C. sativus* L.), melon (*C. melo* L.), watermelon (*C. lanatus*), wax gourd (*B. hispida*), pumpkin (*C. maxima*), zucchini (*C. pepo* L.), silver-seed gourd (*C. argyrosperma*), and bottle gourd (*L. siceraria*). We systematically performed gene structure, conserved motifs, chromosomal location, *cis*-acting elements, and phylogenetic analysis of 59 *YABBY* genes in the eight cucurbit species. The evolutionary relationship of *YABBY* genes between cucumber and other cucurbit species was explored. Finally, the expression patterns of *YABBY* genes in cucumber were investigated in various organs/tissues and different development stages of ovary/fruit. Our results will provide valuable clues for the function identifications of *YABBY* genes in Cucurbitaceae.

## 2. Materials and Methods

### 2.1. Gene Identification and Chromosomal Locations

The YABBY domain (PF04690) was downloaded from the Pfam protein families database [46] (http://pfam.xfam.org/ (accessed on 6 October 2021)) and was used to identify *YABBY* genes in eight cucurbit species by HMMER 3.0 software (E-value < 1 × 10^‒10^). All predicted YABBY sequences were further manually examined to confirm the conserved C2C2 zinc finger domain at N-terminus and YABBY domain at C-terminus using CDD [47] (https://www.ncbi.nlm.nih.gov/cdd (accessed on 6 October 2021)) and SMART [48] (http://smart.embl-heidelberg.de (accessed on 6 October 2021)). The *YABBY* genes of each species were mapped on chromosomes by the online software MapGene2Chrom [49] (http://mg2c.iask.in/mg2c_v2.0/ (accessed on 6 October 2021)). The physicochemical properties, including molecular weights (MW) and isoelectric points (pI), were predicted with the ProtParam tool on the ExPASy server [50] (https://web.expasy.org/protparam/ (accessed on 6 October 2021)). The genome data for eight species in Cucurbitaceae were downloaded from Cucurbit Genomics Database (http://cucurbitgenomics.org/ (accessed on 6 October 2021)) and Arabidopsis protein data (Araport11 protein lists) was downloaded from TAIR (https://www.arabidopsis.org/ (accessed on 6 October 2021)).

### 2.2. Phylogenetic and Gene Duplication Analysis

Multiple sequence alignment of all identified YABBYs in Cucurbitaceae was carried out using ClustalW, and a phylogenetic tree was generated by neighbor-joining (NJ) method with default parameters: bootstrap method setting to 1000, Poisson model, and complete deletion in MEGA 11. The tree was visualized and optimized via Interactive Tree Of Life (iTOL) (https://itol.embl.de/ (accessed on 7 October 2021)). To explore the gene duplication events, the collinearity analysis was performed with Multiple Collinearity Scan toolkit (MCScanX) [51].

### 2.3. Gene Structure Analysis and Conserved Motif Identification

All of the identified *YABBY* gene structures were analyzed by Gene Structure Display Server (GSDS) [52] (http://gsds.gao-lab.org/ (accessed on 7 October 2021)). The MEME online program [53] (https://meme-suite.org/meme/tools/meme (accessed on 7 October 2021)) was employed to predict the motifs within the 59 Cucurbitaceae YABBY protein sequences. 

### 2.4. Cis-Regulatory Elements Analysis 

The promoter sequences (2000 bp upstream of ATG) of 59 *YABBY* genes were extracted from genome sequences of eight cucurbit species by TBtools software [54]. The *cis*-regulatory elements in promoter region were analyzed using the online PlantCARE database [55] (http://bioinformatics.psb.ugent.be/webtools/plantcare/html/ (accessed on 8 October 2021)).

### 2.5. Plant Materials

The cucumber cultivar (“Xintaimici”) was grown in a greenhouse in Beijing, China. Roots and tender stems of two-week-old seedlings, the third true leaves, tender tendrils, male and female buds at 8 DBF (days before flowering), and ovaries/fruits at different growth stages were selected as samples, frozen in liquid nitrogen, and stored at −80 °C.

### 2.6. RNA Extraction and qRT-PCR Analysis

The sample RNA was extracted using the Quick RNA Isolation Kit (Huayueyang, Beijing, China). The FastKing gDNA Dispelling RT SuperMix (TianGen Biotech, Beijing, China) was applied to synthesize the first-strand cDNA with the extractive RNA template. qRT-PCR was performed using the UltraSYBR Mixture (Low ROX) (Cwbio, Beijing, China) on an Applied Biosystems 7500 real-time PCR system (Applied Biosystems, Foster City, CA, USA). The *UBIQUITIN EXTENSION PROTEIN* (*UBI-EP*) gene [56] was used as a reference gene. Three biological and three technical replicates were carried out for expression dynamics analysis. The significant differences were analyzed by Student’s *t*-tests (*p* ≤ 0.05). The primers were listed in Table S6.

### 2.7. Transcriptome Analysis of YABBY Genes in Cucumber

For the expression patterns of *CsYABBY* genes at 4 DBF, ovaries from two near isogenic lines with different fruit lengths were obtained from publicly available transcriptomic data, which were downloaded from Gene Expression Omnibus (GEO) and analyzed to reveal the genes and gene networks that regulate fruit length in cucumber (GSE60346) [57]. Clean tags were remapped to the cucumber v3 genome sequence (http://cucurbitgenomics.org (accessed on 20 October 2021)) by Hisat2, and the TPM values were recalculated. Every line had two biological replicates. The *p*-value ≤ 0.05 and fold-change ≥ 1.5 were used to define differential expression genes. The expression pattern of the *YABBY* genes was shown on a heatmap using TBtools software [54].

## 3. Results

### 3.1. Identification of YABBY Genes in Eight Cucurbit Species and Their Chromosomal Distribution

To identify YABBY members in Cucurbitaceae, we performed a Hidden Markov Model (HMM) search using YABBY domain (PF04690) across eight cucurbit species. Based on a further confirmation of conserved C2C2 domain and YABBY domain by CDD and SMART analysis, 59 *YABBYs* were finally obtained in Cucurbitaceae, including 8 from cucumber (*C. sativus* L.), 4 from melon (*C. melon* L.), 9 from watermelon (*C. lanatus*), 5 from wax gourd (*B. hispida*), 11 from pumpkin (*C. maxima*), 9 from zucchini (*C. pepo* L.), 10 from silver-seed gourd (*C. argyrosperma*), and 3 from bottle gourd (*L. siceraria*) (Table 1 and Table S1). These gene coding sequence lengths varied from 471 to 1179 bp and the encoded protein length ranged from 156 to 392 amino acids along with the predicted protein molecular weight (MW) ranging from 17.05 to 43.64 KDs. The isoelectric point (pI) values of the YABBYs were 4.23 to 9.62 (Table S2).

Next, we mapped the *YABBY* genes on chromosomes of seven cucurbit species (except silver-seed gourd with a lower quality genome draft), respectively. The *YABBY* genes of each cucurbit species were randomly distributed on their chromosomes and distribution results were shown in Figure S1. For example, the chromosome 1, 2, 3, 5, and 6—but not chromosome 4 and 7—harbored the eight *YABBY* genes in cucumber, the nine *YABBYs* were distributed on chromosome 1, 2, 5, 6, 8, 10, and 11 other than chromosome 3, 4, 7, and 9 in watermelon (Figure S1).

### 3.2. Evolutionary Relationship and Synteny Analysis of YABBYs in Cucurbitaceae

**Figure 1:**
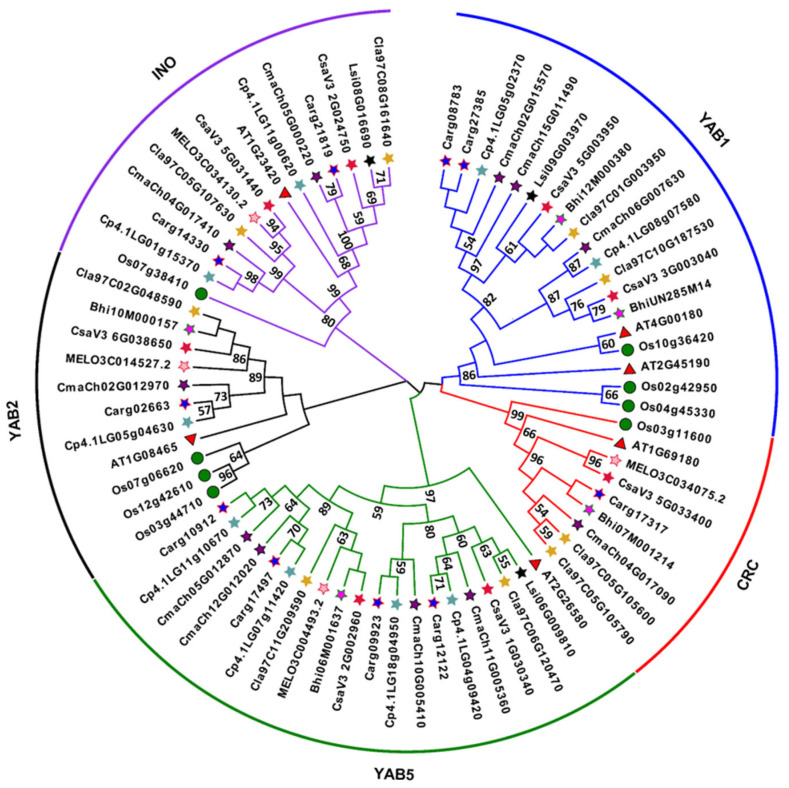
Phylogenetic tree of the YABBY proteins from Arabidopsis, rice, and eight cucurbit species. Red star, red star filled with pink color, yellow star, green star filled with light purple color, black star filled with dark purple color, bluish grey star, red star filled with blue color, black star, black triangle filled with red color and black circle filled with green color represent the YABBYs from cucumber, melon, watermelon, wax gourd, pumpkin, zucchini, silver-seed gourd, bottle gourd, Arabidopsis, and rice, respectively. Cs, cucumber (*C. sativus* L.); MELO, melon (*C. melo* L.); Cla, watermelon (*C. lanatus*); Bhi, wax gourd (*B. hispida*); Cma, pumpkin (*C. maxima*); Cp, zucchini (*C. pepo* L.); Carg, silver-seed gourd (*C. argyrosperma*); Lsi, bottle gourd (*L. siceraria*); AT, Arabidopsis (*Arabidopsis thaliana*); Os, rice (*Oryza sativa* L.).

To better explore the evolutionary relationship of YABBYs, six Arabidopsis YABBYs, eight rice YABBYs and 59 YABBY members in Cucurbitaceae were used to construct an evolutionary tree using MEGA 11 with ClustalW and NJ methods. As reported in Arabidopsis, the Cucurbitaceae YABBYs were also divided into five subfamilies, YAB1, YAB2, CRC, INO, and YAB5 (Figure 1 and Table 1). The YAB1 and YAB5 subfamilies had the larger numbers of YABBYs, in which the YAB1 subfamily contained 14 members while YAB5 subfamily contained 19 members. The two subfamilies of YAB2 and CRC shared the smallest YABBYs with seven members, respectively. The four cucurbit species cucumber, watermelon, pumpkin, and silver-seed gourd YABBYs could be divided into all five subfamilies. However, the other four cucurbit species lack one or two subfamilies, such as melon lacking YAB1 subfamily, wax gourd lacking INO subfamily, zucchini lacking CRC subfamily, and bottle gourd lacking CRC and YAB2 subfamilies (Figure 1 and Table 1). Taken together, these results suggested that there are evolutionary splits and diversifications of YABBYs among different cucurbit species. 

**Figure 2:**
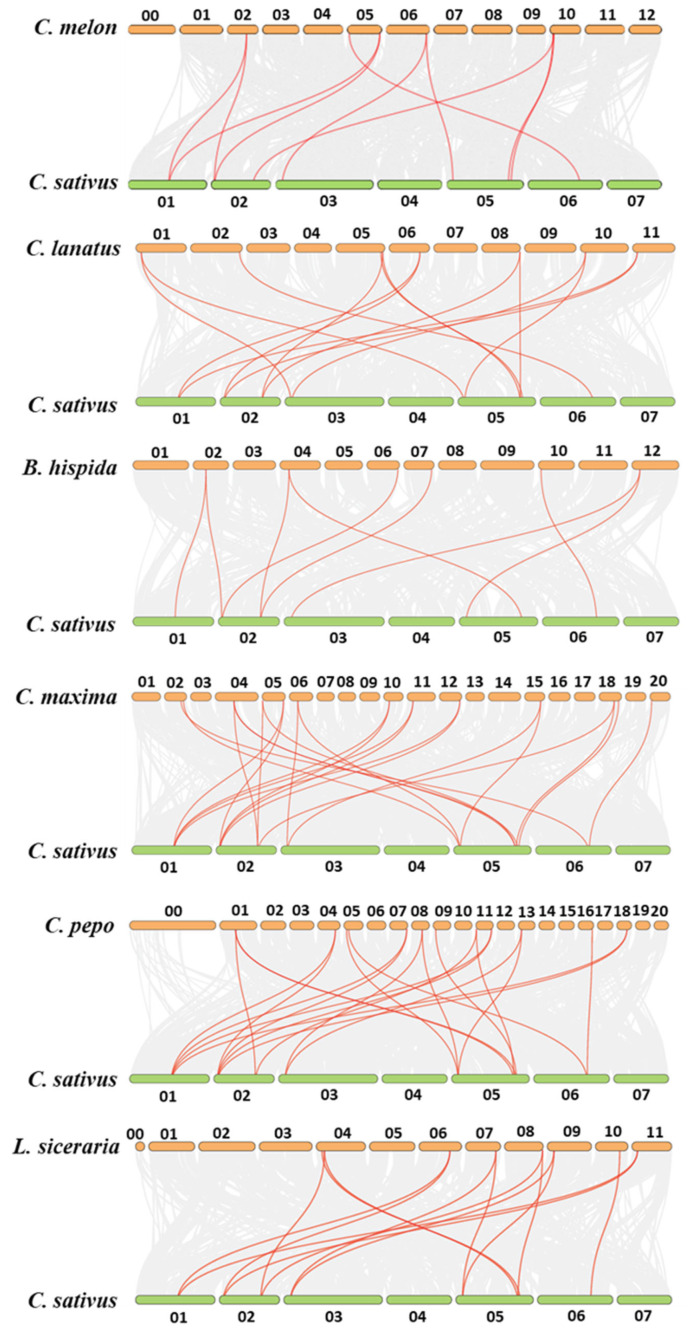
Synteny analysis of *YABBY* genes between cucumber and melon, watermelon, wax gourd, pumpkin, zucchini, and bottle gourd. The synteny gene pairs are highlighted in the red lines.

Generally, gene duplications contribute to novel gene function generation and gene family expansion [58]. Here, we performed a synteny analysis of *YABBYs* between cucumber and other cucurbit species (except silver-seed gourd with a lower quality genome draft) by MCScanX. The collinear gene pairs between cucumber and melon, wax gourd, watermelon, bottle gourd, pumpkin, and zucchini were 10, 9, 14, 14, 19, and 21, respectively (Figure 2 and Table S3). The all eight *CsYABBYs* showed a syntenic relationship (at least two syntenic gene pairs of each *CsYABBY* gene) with zucchini *YABBYs*, 75% of *CsYABBYs* (except *CsaV3_5G033400* and *CsaV3_6G038650* possessing one syntenic gene pair, respectively) shared two, or more than two, syntenic pairs with the *YABBYs* in watermelon, pumpkin, and bottle gourd (Figure 2 and Table S3), indicating that the *YABBY* genes in cucumber, watermelon, pumpkin, bottle gourd, and zucchini evolved from the same ancestral gene. In addition, six *YABBY* genes had only one syntenic gene pair between cucumber and melon, respectively. However, *CsaV3_1G030340* and *CsaV3_2G002960* were sisters to two melon *YABBY* genes, respectively. The same case was observed between cucumber and wax gourd (only *CsaV3_2G002960* and *CsaV3_2G024750* have two sisters in wax gourd, respectively) (Figure 2 and Table S3), suggesting that there are no apparent gene family expansion events between cucumber and melon/wax gourd. Notably, when we identified *YABBY* genes, the truncated genes, lacking either a C2C2 domain and/or a YABBY domain, were excluded. This is possibly responsible for why the final number of *YABBY* genes of each cucurbit species (except cucumber and watermelon) is less than that obtained by synteny analysis (Table S3). 

### 3.3. Gene Structure and Conserved Motifs Analysis of YABBYs in Cucurbitaceae

**Figure 3:**
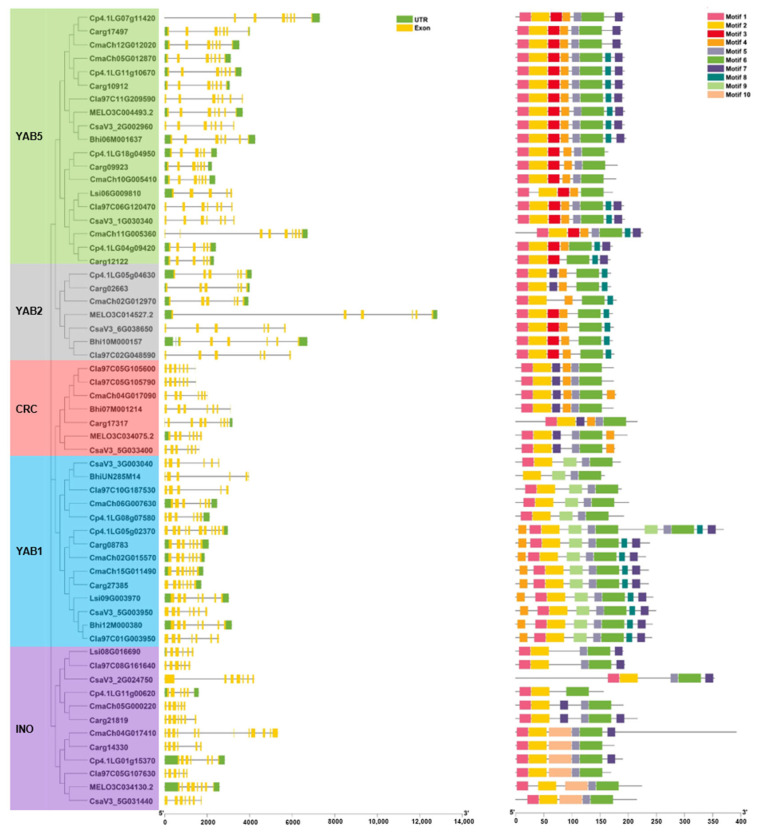
Phylogenetic clustering, conserved motifs, and gene structure of *YABBY* genes from eight cucurbit species. Left panel: unrooted phylogenetic tree of YABBY proteins. Light green, gray, light red, blue and purple part represent YAB5, YAB2, CRC, YAB1, and INO subfamilies, respectively. Middle panel: gene structure of *YABBY* genes. Untranslated regions and exons are indicated by green boxes and yellow boxes, respectively. Right panel: the conserved motifs of YABBYs are represented by different colored boxes.

The evolutionary history of gene families can be reflected by gene structural diversity [59]. Thus, we analyzed the gene structure that are characterized by the exon–intron organization of *YABBYs* open reading fame (ORF) from eight cucurbit species to explore the *YABBYs* evolution in Cucurbitaceae. An unrooted evolutionary tree was conducted by MEGA 11 and used for distinguishing different YAB subfamilies. As shown in Figure 3 and Table S2, the 59 *YABBYs* contained exon numbers ranging from 5 to 12 and intron number varying from 4 to 11. Among them, all YAB2 subfamily members had six exons and five introns, most members of the CRC subfamily (except *Carg17317* with nine exons and eight introns in silver-seed gourd) shared seven exons and six introns (Figure 3 and Table S2). However, we found that the INO subfamily and the two larger subfamilies, YAB1 and YAB5, harbor different numbers of exon and intron, 5 to 11 exons and 4 to 10 introns in the YAB1 subfamily, 5 to 9 exons and 4 to 8 introns in the YAB5 subfamily, and 6 to 12 exons and 5 to 11 introns in the INO subfamily (Figure 3 and Table S2). 

**Figure 4:**
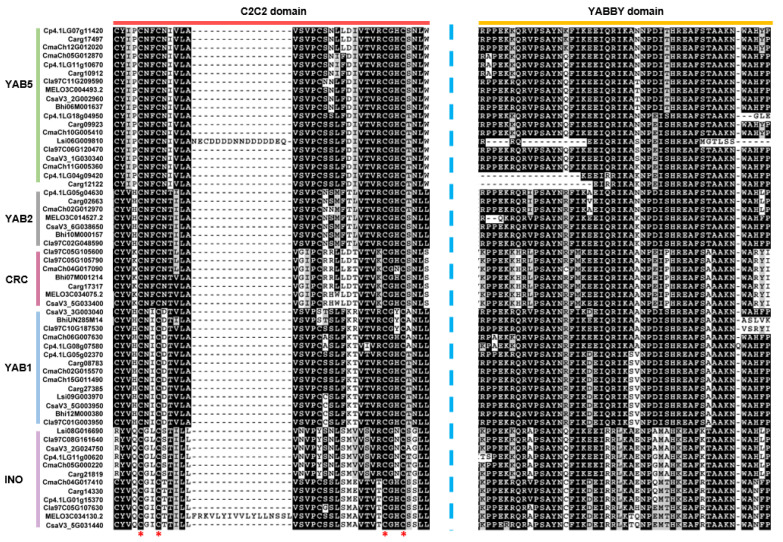
The conserved C2C2 domain and YABBY domain of 59 YABBY proteins from eight cucurbit species. The typical amino acid residues within C2C2 domain are indicated with red asterisks.

The MEME analysis was carried out to predict the conserved motifs of protein sequence to better understand the conservation and diversification of YABBYs in Cucurbitaceae. We observed that the YABBYs in same subfamily share highly similar motif compositions (Figure 3 and Figure S2). For instance, motif 9 was unique to YAB1 subfamily, whereas motif 5 was lacking in YAB2 subfamily. Specially, motif 10 only existed in a subgroup of INO subfamily (Figure 3). These results suggested the possible functional diversification of YABBYs across Cucurbitaceae. Finally, given that the YABBYs are plant-specific proteins containing conserved C2C2 domain and YABBY domain, we aligned the 59 YABBY protein sequences. The alignment results exhibited that all 59 YABBYs contain the two conserved domains (Figure 4), indicating the conservation in Cucurbitaceae YABBYs evolution.

### 3.4. Cis-Acting Elements Analysis of Cucurbitaceae YABBY Genes Promoter Regions

**Figure 5:**
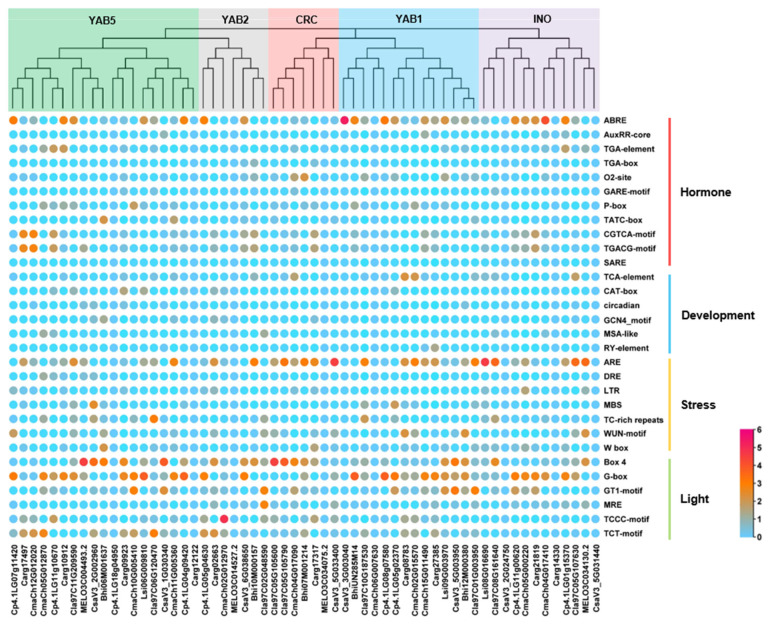
The *cis*-acting elements analysis of 59 *YABBY* genes promoters across Cucurbitaceae. The numbers of *cis*-acting elements are shown in a heatmap.

The 2000 bp upstream sequences from the transcription start site of 59 *YABBYs* were extracted by TBtools from the genome of the eight cucurbit species and used to analyze the *cis*-acting elements in these promoters using the online PlantCARE database. The predicted *cis*-acting elements were mainly associated with phytohormone response, plant development, stress, and light responses (Figure 5 and Table S4). We noted that the light response elements exist in most *YABBYs* promoters. The phytohormone response elements mainly involved in abscisic acid (ABRE) and methyl jasmonate (CGTCA-motif, TGACG-motif) were identified, while fewer response elements associated with auxin (TGA-element), cytokinin (O2-site), gibberellin (P-box), and salicylic acid (TCA-element) were discovered. Moreover, the stress response elements—such as TC-rich repeats for defense and stress, MBS for drought stress, and LTR for low temperature stress—were also found. Notably, we detected some development-related elements but with fewer numbers, including CAT-box (meristem), circadian (circadian control), GCN4_motif (endosperm), MSA-like (cell cycle), and RY-element (seed-specific) (Figure 5 and Table S4). Taken together, these results suggested possible roles of *YABBYs* in plant development, stress, and phytohormone responses across Cucurbitaceae.

### 3.5. Expression Pattern of YABBYs in Cucumber

**Figure 6:**
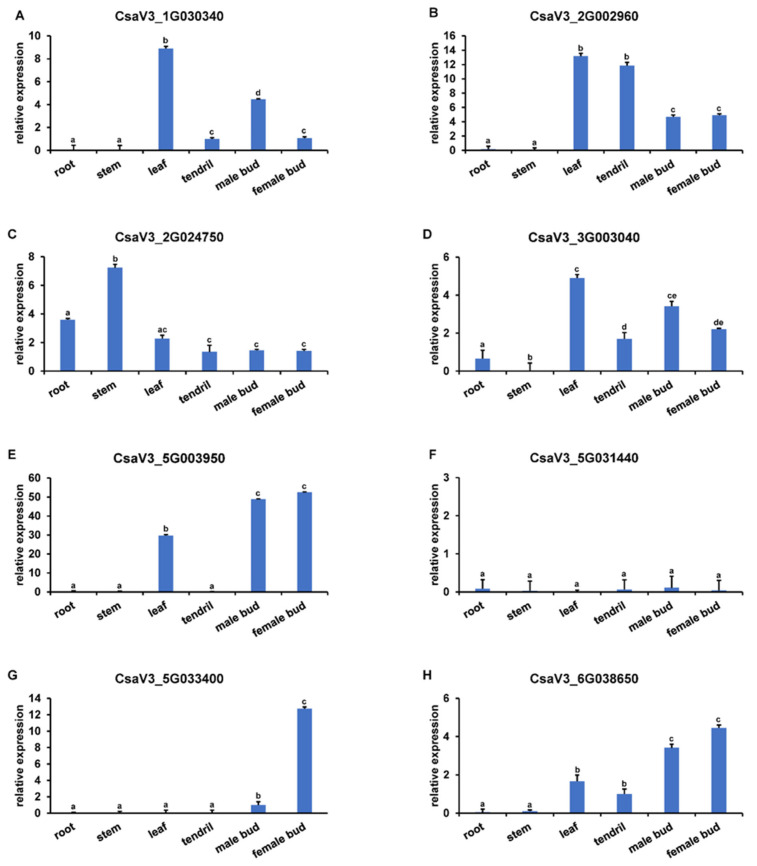
The expression patterns of *CsYABBY* genes in different tissues of cucumber. (**A**–**H**) Tissues-specific of *CsYABBY* expression was examined in cucumber by qRT-PCR. Values are means ± SD of three biological replicates.

**Figure 7:**
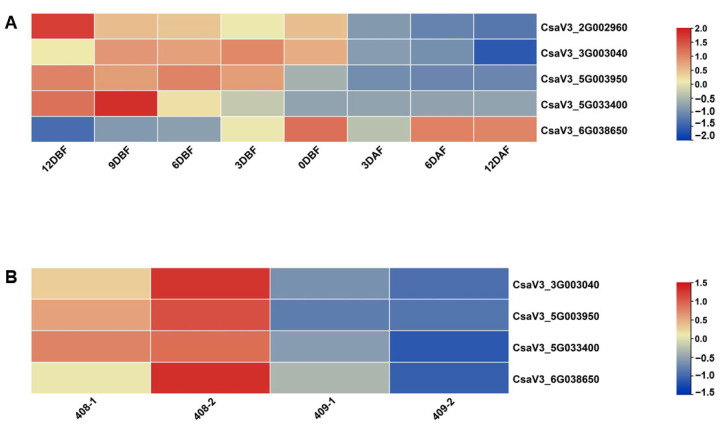
The expression patterns of *CsYABBY* genes in different development stages of ovary/fruit of cucumber. (**A**) The relative expression levels of *CsYABBYs* at different development stages of ovary/fruit. (**B**) The TPM values of *CsYABBYs* at 4 DBF ovary in near-isogenic lines 408 and 409. DBF: days before flowering. DAF: days after flowering. Values are means ± SD of three biological replicates.

To explore the detailed expression profiles of *YABBYs* in Cucurbitaceae, we selected cucumber as a representative species to examine *YABBY* gene expression patterns in different organs including root, stem, leaf, tendril, male bud, female bud, and different development stages of ovary/fruit. As shown in Figure 6, the *CsYABBYs* exhibited various expression patterns in test materials. The four genes *CsaV3_1G030340*, *CsaV3_2G002960*, *CsaV3_3G003040*, and *CsaV3_5G003950* had high expression levels in leaf. We noticed that *CsaV3_2G002960* also expressed higher in tendrils and *CsaV3_2G024750* shows high expression level in stems. Moreover, we found that *CsaV3_5G003950* and *CsaV3_5G033400* are highly expressed in floral organs. In addition, *CsaV3_2G002960*, *CsaV3_3G003040*, and *CsaV3_6G038650* also had higher expression levels in male and female buds. However, the expression of *CsaV3_5G031440* was mostly undetectable in all organs (Figure 6). Given the higher expression levels of *CsaV3_2G002960*, *CsaV3_3G003040*, *CsaV3_5G003950*, *CsaV3_5G033400* and *CsaV3_6G038650* in floral organs, we further explore the expression patterns of these five genes in different development stages of ovary/fruit. We found that *CsaV3_2G002960*, *CsaV3_3G003040*, *CsaV3_5G003950*, and *CsaV3_5G033400* are mainly expressed at all ovary development stages (12, 9, 6, 3, and 0 DBF) but not fruit development stages, whereas *CsaV3_6G038650* shows high expression at all fruit development stages (3, 6, and 12 DAF) other than ovary development stages (Figure 7A). The similar expression patterns were also observed in an RNA-seq data wherein a transcriptome analysis of 4 DBF ovary from two near-isogenic lines 408 and 409 was performed [57] (Figure 7B and Table S5). Four *YABBY* genes (except *CsaV3_2G002960* with no differential expression) were higher expressed in line 408 which is characterized by long fruit compared to the line 409 which has a short fruit [57] (Figure 7B and Table S5). Hence, the results indicated that the *YABBYs* may play various and important roles during plant growth and development in cucumber, such as leaf, tendril, stem, male flower, and ovary/fruit development.

## 4. Discussion

The YABBYs, plant-specific transcription factors, play significant roles during different plant development processes [12,13]. So far, it is well known that Cucurbitaceae—such as cucumber, melon, watermelon, and pumpkin—are important horticultural crops with global economic value [31]. However, little is known about the characteristics and functions of *YABBYs* across Cucurbitaceae, except some studies of cucumber [44,45].

Recently, the whole-genome sequences of eight cucurbit species—including cucumber, melon, watermelon, wax gourd, pumpkin, zucchini, silver-seed gourd, and bottle gourd—were released or updated [34,60,61,62,63,64,65,66], providing a useful strategy to deepen our understanding of genome-wide identification of YABBY family in Cucurbitaceae. In this study, we identified and characterized the *YABBY* genes in Cucurbitaceae based on these whole-genome sequences. Totally, we obtained 3, 4, 5, 8, 9, 9, 10, and 11 *YABBY* genes in bottle gourd, melon, wax gourd, cucumber, watermelon, zucchini, silver-seed gourd, and pumpkin, respectively (Table 1 and Table S1). Like Arabidopsis *YABBY* genes [10,11], we found that the *YABBY* genes in eight cucurbit species are also divided into five subfamilies, YAB1, YAB2, CRC, INO, and YAB5 (Figure 1 and Table 1). Notably, compared to Arabidopsis which has six *YABBY* genes [1,2,3], the number of *YABBY* genes in bottle gourd, melon, and wax gourd was less than six but more than six in the other five cucurbit species (Table 1 and Table S1). We speculated that the differences of *YABBY* gene numbers among different cucurbit species may be explained by gene duplication or loss during evolutionary process, as the segment and tandem duplications contribute to the expansion of gene family [58]. The synteny analysis results showed that there are many more syntenic gene pairs between cucumber and watermelon, bottle gourd, pumpkin, and zucchini (Figure 2 and Table S3). In addition, we observed that an obvious expansion of INO and YAB5 subfamilies among Cucurbitaceae, which possibly is owing to much more orthologs within the two subfamilies in watermelon, pumpkin, and zucchini except bottle gourd (Figure 1 and Table 1). These results suggested that gene duplication may contribute to the more *YABBY* genes in these three cucurbit species except bottle gourd. However, for melon and wax gourd which had less *YABBY* genes (Table 1 and Table S1), we noticed that 75% (6 of 8) of *CsYABBY* genes share only one syntenic gene pair between cucumber and melon/wax gourd (Figure 2 and Table S3), and the loss events also exist in melon and wax gourd, including no YAB1 subfamily in melon and no INO subfamily in wax gourd (Figure 1 and Table 1). Furthermore, although more syntenic gene pairs between cucumber and bottle gourd were found (Figure 2 and Table S3), bottle gourd contained only three *YABBY* genes and lacked CRC and YAB2 subfamilies (Figure 1 and Table 1). Since the *YABBY* gene contains a conserved C2C2 domain and a conserved YABBY domain, the truncated genes, which lack either a C2C2 domain and/or a YABBY domain, were excluded when we identified the *YABBY* genes, which might account for the final number of identified *YABBY* genes in Cucurbitaceae (except cucumber and watermelon) being lower than that obtained by synteny analysis and one and/or two YABBY subfamilies are lacking in melon, wax gourd, zucchini, and bottle gourd. Possibly due to the low quality of silver-seed gourd genome draft, we could not perform the synteny analysis between cucumber and silver-seed gourd. Hence, further studying is recommended to explore whether lower quality of genome draft or gene loss events during evolutionary process are the cause of fewer *YABBY* genes and the lacking of some subfamilies in these cucurbit species.

To better understand the conservation and diversification of YABBYs in Cucurbitaceae, we performed an analysis of gene structures and conserved motifs/domains. The 59 YABBYs shared a conserved C2C2 domain and a conserved YABBY domain (Figure 4 and Table S1). However, there were some differences among different subfamilies. For instance, all YAB2 subfamily members had six exons and five introns, whereas CRC subfamily (except *Carg17317* with nine exons and eight introns in silver-seed gourd) members contained seven exons and six introns. YAB2 and CRC subfamilies commonly contained motif 1, motif 2, motif 4, and motif 6; meanwhile, the YAB2 subfamily had motif 8 but lost motif 5, whereas the CRC subfamily had motif 5 but lacked motif 8 (Figure 3). These results hinted that the similar characteristics of *YABBY* genes within the same subfamily may indicate conserved function, and the differences of *YABBY* gene characteristics among various subfamilies possibly contribute to functional differentiation across Cucurbitaceae. Generally, transcription factors bind to specific *cis*-acting elements of targeted genes to regulate their expression. The *cis*-acting elements in promoters of the 59 *YABBY* genes suggested that they may be involved in plant development, phytohormone, stress, and light responses in Cucurbitaceae.

Importantly, the expression patterns of *CsYABBY* genes in cucumber were analyzed to explore the potential functions of different YABBY subfamilies across Cucurbitaceae. Previous reports found that *YAB1* (*FIL*)/*YAB3*, *YAB2*, and *YAB5* are associated with leaf and floral organ development in Arabidopsis and rice [2,14,16,18,19,29,30,67], the similar higher expression levels of their homologous genes (except *YAB2* homolog) *CsaV3_5G003950*, *CsaV3_3G003040*, *CsaV3_2G002960*, and *CsaV3_1G030340* in leaf and floral organs suggested the functional conservation of these genes between cucumber, Arabidopsis, and rice (Figure 6). Meanwhile, the special expression of *CsaV3_5G033400*, the homolog of *CRC* which functions in carpel development in Arabidopsis [1,20,21] and rice [23], in female bud hinted the underlying role in carpel development of cucumber (Figure 6). However, we observed that the *YAB2* homolog *CsaV3_6G038650* expression level is higher only in floral organs, but not leaf or other tissues. *CsaV3_2G002960* was also highly expressed in tendrils. Additionally, there are two homologs of *INO* in cucumber. According to the tissue-specific feature with low levels in various tissues of *INO* expression in Arabidopsis [3], we detected almost no expression of *CsaV3_5G031440* in cucumber tissues. However, the other gene *CsaV3_2G024750* were expressed in different tissues, especially in stem (Figure 6), although *CsaV3_2G024750* (gene ID in paper: *Csa011583*) was reported in involved with integument of ovules but not with functional verification [44]. Thus, the different or specific expression patterns of these four genes suggested their function differentiations in cucumber. Further studies are needed to verify our speculation by their functional identifications. 

Furthermore, four genes—*CsaV3_2G002960*, *CsaV3_3G003040*, *CsaV3_5G003950*, and *CsaV3_5G033400*—showed higher expression levels at all ovary development stages but not in fruits (Figure 7A), suggesting their possible roles in ovary development of cucumber. Particularly, *CsaV3_5G003950*—the homolog of *YAB1*—had very high transcriptional level and thus might mainly regulate ovary development of cucumber, this is in line with the function of *YAB1* which is involved in abaxial cell type specification in leaves and fruits in Arabidopsis [2,14,16]. In rice, the YAB1 clade genes—*TOB1*, *TOB2*, and *TOB3*—are required to maintain proper function of the spikelet and branch meristems [29,30]. It is likely that the function of YAB1 clade genes has been conserved between cucumber, rice, and Arabidopsis. For the *CRC* homolog in cucumber, *CsaV3_5G033400*, was significantly expressed in the early development stage of ovary at 12DBF and 9DBF (Figure 7A), indicating its possible role in carpel development of cucumber and is similar with CRC function involved in carpel development in Arabidopsis [1,20,21] and rice [23]. Interestingly, we noted that the *YAB2* homolog *CsaV3_6G038650* is more highly expressed in fruit than ovary (Figure 7A). Although *YAB2* is involved in polarity development of leaf and flower [2], the function in regulating fruit development is not proved in Arabidopsis. The specific expression pattern of *CsaV3_6G038650* in fruit implied its prominent function in cucumber fruit development. Coincidentally, *fas*—a YABBY-like transcription factor homologous to Arabidopsis *YAB2*—regulated fruit development by controlling carpel number in tomato, resulting in an extreme fruit size [68]. Additionally, in cereals, *SH1* or *ObSH3*—which are closely related to Arabidopsis *YAB2*—is required for seed shattering during domestication [26,27]. These results suggested the functional differentiation of *YAB2* gene between cucumber, tomato, cereals, and Arabidopsis, and *CsaV3_6G038650* is a possibly major regulatory factor of fruit development in cucumber. Taken together, the results implied that the *YABBY* genes possibly play vital roles in many aspects of plant growth and development in cucumber—such as leaf, tendril, and ovary/fruit—which will contribute to the applications of *YABBY* genes in breeding of cucumber, even Cucurbitaceae.

## 5. Conclusions

In this study, 59 *YABBY* genes were identified from eight cucurbit species. A systematic characterization study was performed for chromosomal location, gene structure, conserved motifs, *cis*-acting elements, evolutional relationship, and gene duplication. The evolutionary relationship showed that the *YABBY* genes from Cucurbitaceae are classified into five subfamilies. Gene duplication events occurred between cucumber and watermelon, bottle gourd, pumpkin, and zucchini, which contributes to *YABBY* gene family expansions in these four cucurbit species. However, we have not confirmed whether or not low quality of genome draft or gene loss events are the cause of fewer *YABBY* genes in bottle gourd, melon, and wax gourd—further verification is required. The expression patterns of most *YABBY* genes in cucumber were similar with that in Arabidopsis and rice, indicating the function conservation of these *YABBY* genes among Cucurbitaceae. However, the different expression patterns of several *CsYABBY* genes are needed to illuminate by their function verifications. Importantly, we identified *CsaV3_6G038650* as a potential regulatory factor of fruit development in cucumber. In conclusion, our study provided a foundation for further research on *YABBY* gene functions which will facilitate breeding in Cucurbitaceae.

## Figures and Tables

**Table 1:** Classification of the *YABBY* gene family in eight cucurbit species.

Species	Group					Total
	YAB1	CRC	INO	YAB2	YAB5	
*C*. *sativus* L.	2	1	2	1	2	8
*C*. *melo* L.	0	1	1	1	1	4
*C. lanatus*	2	2	2	1	2	9
*B. hispida*	2	1	0	1	1	5
*C. maxima*	3	1	2	1	4	11
*C. pepo* L.	2	0	2	1	4	9
*C. argyrosperma*	2	1	2	1	4	10
*L. siceraria*	1	0	1	0	1	3

## Data Availability

Data used in this study are presented in the article or supplementary information.

## Supplementary Materials

The following are available online at https://www.mdpi.com/article/10.3390/genes13030467/s1. Figure S1: Chromosomal locations of *YABBY* genes in the genome of seven cucurbit species; Figure S2: Analysis of conserved motifs in YABBY proteins of eight cucurbit species; Table S1: List of *YABBY* genes identified in cucumber, melon, watermelon, wax gourd, pumpkin, zucchini, silver-seed gourd, and bottle gourd; Table S2: Information of *YABBY* genes identified in eight cucurbit species; Table S3: Orthologous relationships of *YABBY* genes between cucumber and other six cucurbit species; Table S4: *Cis*-acting elements of *YABBY* genes promoters in cucumber, melon, watermelon, wax gourd, pumpkin, zucchini, silver-seed gourd, and bottle gourd; Table S5: The TPM values of YABBY family genes in cucumber by RNA-Seq analysis; Table S6: Primers of cucumber *YABBY* genes used in qRT-PCR.
