# Supplementary material for: Genome-Wide Identification of YABBY Gene Family in Cucurbitaceae and Expression Analysis in Cucumber (Cucumis sativus L.)
Source: Genes (Basel). 2022 Mar 7;13(3):467. doi: 10.3390/genes13030467 (PMC8953090; doi:10.3390/genes13030467)
Supplement: Supplementary file 1 [file genes-13-00467-s001.zip › supplementary files-revised (Round 2)/suplement figures.pptx]

## Slide 1
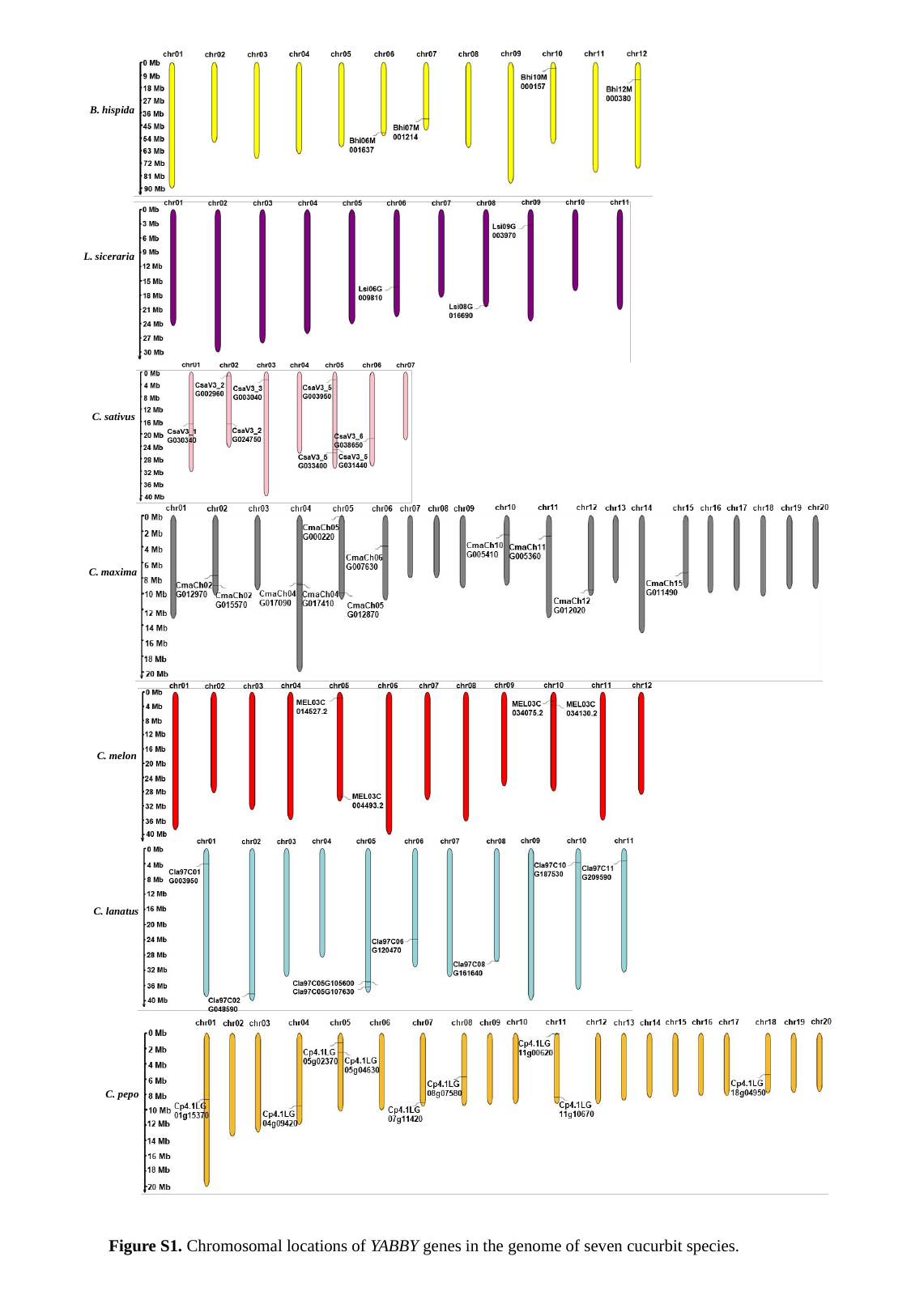

B. hispida
L. siceraria
C. sativus
C. maxima
C. melon
C. lanatus
C. pepo
Figure S1. Chromosomal locations of YABBY genes in the genome of seven cucurbit species.

## Slide 2
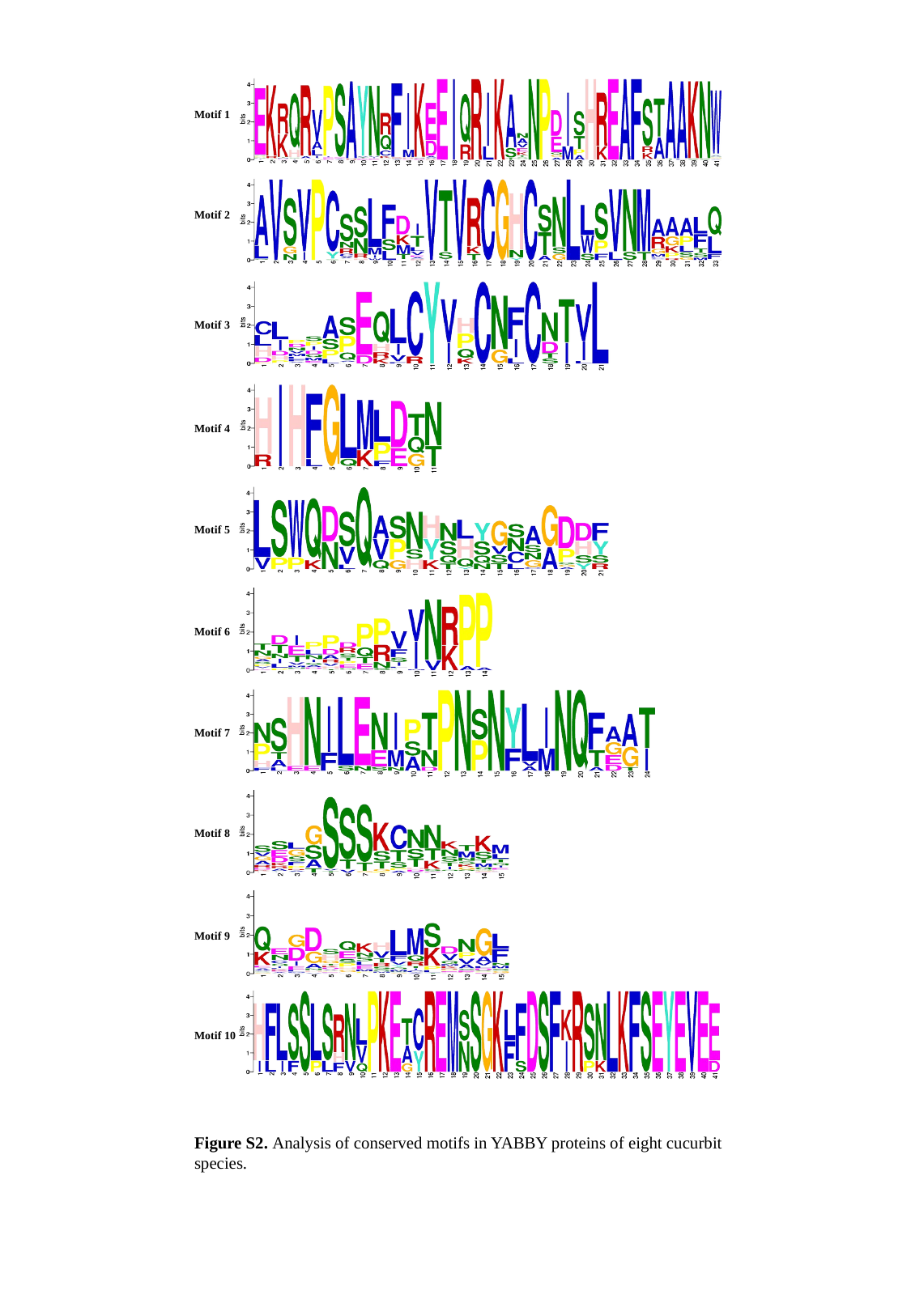

Motif 1
Motif 2
Motif 3
Motif 4
Motif 5
Motif 6
Motif 7
Motif 8
Motif 9
Motif 10
Figure S2. Analysis of conserved motifs in YABBY proteins of eight cucurbit species.
